# Using a novel psychosocial group intervention to improve adaption, coping and mental health outcomes following dysvascular limb amputations: A feasibility study

**DOI:** 10.33137/cpoj.v8i1.45122

**Published:** 2025-05-24

**Authors:** R.J Steinberg, L.R Robinson, O Kachmarchuk, S Jankey, S Posa, A.L Mayo, M Simon, A Kiss, C MacKay, R Simpson, M.B Wasilewski, S Dilkas, S.L Hitzig

**Affiliations:** 1 St. John's Rehab Research Program, Sunnybrook Research Institute, Sunnybrook Health Sciences Centre, Toronto, Canada.; 2 Consultation/Liaison Psychiatry, Adult Psychiatry and Health Systems, Sunnybrook Health Sciences Centre, Toronto, Canada.; 3 Division of Physical Medicine & Rehabilitation, Department of Medicine, Sunnybrook Health Sciences Centre, Toronto, Canada.; 4 Physical Medicine and Rehabilitation, Temerty Faculty of Medicine, University of Toronto, Toronto, Canada.; 5 Rehabilitation Sciences Institute, Temerty Faculty of Medicine, University of Toronto, Toronto, Canada.; 6 West Park Healthcare Centre, University Health Network, Toronto, Canada.; 7 St. John's Rehab Hospital, Sunnybrook Health Sciences Centre, Toronto, Canada.; 8 Institute of Health Policy, Management and Evaluation, University of Toronto, Toronto, Canada.; 9 Department of Physical Therapy, Temerty Faculty of Medicine, University of Toronto, Toronto, Canada.; 10 School of Rehabilitation Therapy, Queen's University, Kingston, Canada.; 11 Institute of Health and Wellbeing, University of Glasgow, Glasgow, United Kingdom.; 12 Toronto Rehabilitation Institute, University Health Network, Toronto, Canada.; 13 Department of Occupational Science and Occupational Therapy, Temerty Faculty of Medicine, University of Toronto, Toronto, Canada.; 14 Dalla Lana School of Public Health, Clinical Public Health Division, University of Toronto, Toronto, Canada.

**Keywords:** Psychotherapy, Amputation, Group, Amputees, Feasibility Studies, Mental Health, Rehabilitation, Dysvascular, Prosthetics

## Abstract

**BACKGROUND:**

Individuals with lower extremity amputations (LEA) often face high rates of depression and anxiety that hinder their rehabilitation and post-discharge coping. Group therapy is a clinically and cost-effective way to address these mental health challenges, but evidence for its use with LEA inpatients is limited.

**OBJECTIVE:**

To determine the feasibility of a psychosocial group therapy intervention for individuals with dysvascular LEA undergoing inpatient rehabilitation.

**METHODOLOGY:**

This randomized controlled trial randomly assigned dysvascular LEA rehabilitation inpatients into a supportive-expressive group therapy (SEGT) or a treatment as usual (TAU) group. The SEGT intervention, a form of group therapy adapted from outpatient medical settings, consisted of six one-hour sessions held twice weekly over a three-week period. Participants completed baseline, exit and three-month surveys assessing the study's secondary outcomes of SEGT effectiveness on depression, anxiety, coping, body image, health, and community participation. The main outcomes assessed recruitment, survey completion, treatment adherence, and participant retention rates. Interviews and a focus group were completed to obtain feedback on the intervention.

**FINDINGS:**

Twenty-five participants were recruited, with 12 randomly assigned to the SEGT group, and 13 to the TAU group. The average number of sessions attended by SEGT participants was 3.9 (SD = 2.1). The survey completion rates for all participants were 84% (21/25) for the baseline assessment, 64% (18/25) for discharge, and 44% (11/25) for the three-month follow-up. The SEGT group showed a significant improvement in anxiety and depression scores (p = 0.02). SEGT was well-received by participants and staff.

**CONCLUSION:**

The findings suggest a larger pragmatic SEGT trial is feasible, despite a small sample size and implementation challenges during the COVID-19 pandemic, given this study achieved moderate rates of recruitment, retention, and survey completion. Several critical insights were gained on how to optimize an inpatient group therapy intervention for dysvascular LEA populations in rehabilitative settings.

## INTRODUCTION

Lower extremity amputations (LEA) are a debilitating event that can negatively affect an individual's physical and mental health.^1–3^ Although there are many factors that lead to LEA, approximately 80% are dysvascular in etiology and attributed to complications of diabetes and/or peripheral arterial disease.^4–6^ Compared to other limb loss populations (e.g., traumatic etiology), people with dysvascular LEA have been shown to have poorer quality of life,^1^ and higher rates of post-morbid complications including depression, anxiety and impaired body image.^1,7,8^ A comorbid diagnosis of depression is associated with lower prosthetic use, higher perceived vulnerability, and lower self-rated health.^9^

Individuals with LEA undergoing inpatient rehabilitation may receive psychiatric consultations, but individual assessments can be time-consuming and costly.^10,11^ Group therapy is a less-resource intensive mental health intervention, whereby one or more healthcare providers can treat a group of patients simultaneously.^12^ There is some preliminary evidence that group therapy may be beneficial for inpatients.^13–15^ For instance, one randomized clinical trial reported that LEA inpatients experienced improvements in anxiety, depression and body image after participating in group therapy.^15^ Similarly, a three session group therapy intervention for limb loss inpatients of mixed etiologies (trauma, dysvascular), including upper limb amputation, found that group therapy participants showed significantly lower distress levels than participants in the comparison group.^13^ In one study, however, the etiology of the sample was not described,^15^ and the other used a comparison group of discharged patients who were residing in the community.^13^ As such, the variable features of these studies makes generalizability to inpatients with dysvascular LEA somewhat challenging.

To assess the applicability of a group therapy model of care for dysvascular LEA inpatients, the primary aim of this study was to evaluate the feasibility of implementing a novel supportive-expressive group therapy (SEGT) program for inpatients with dysvascular LEA. SEGT is one type of group therapy that has been previously used in patients with medical illness^16–18^ which provides emotional, social and cognitive support. The secondary aim was to assess whether these SEGT sessions had any effect on mental health outcomes, with the expectation that SEGT participants would demonstrate measurable symptomatic improvements compared with those participants assigned to a treatment as usual (TAU) condition.

## METHODOLOGY

This two-armed feasibility trial was conducted between October 2021 and February 2023. A feasibility trial design was selected since there are only two studies^13,15^ regarding the use of group therapy for dysvascular LEA populations in an inpatient rehabilitation setting. The Research Ethics Board at the Sunnybrook Health Sciences Centre approved the study, and it was registered at ClinicalTrials.gov (ID# NCT05082870).

Participants were randomly assigned to either a supportive-expressive group therapy (SEGT) group or to a treatment as usual (TAU) group. It was hypothesized that an inpatient SEGT intervention would be feasible, and that a larger pragmatic trial could be developed as a result. The goal was to recruit 50 inpatients, with 25 randomized to the TAU group and 25 randomized to the SEGT group. To minimize contamination between cohorts, cluster randomization was used to have TAU and SEGT cohorts occur in different months; thereby minimizing the likelihood that SEGT participants would overlap with TAU participants. This was done in order to reduce the possibility that participants from different cohorts could discuss the study conditions with each other. A blinding protocol was followed to create the randomization using an online randomizer (https://www.randomizer.org/) consisting of six blocks (three for SEGT; three for TAU).

### Participants

Participants were recruited from the Cardiac and Amputee unit at St. John's Rehab, Sunnybrook Health Sciences Centre (Ontario, Canada). The inclusion criteria were:

- Adult inpatient aged 18 years or older;
- Have a dysvascular LEA;
- No clinical suspicion of cognitive impairments or a severe mental health diagnosis (e.g., schizophrenia, dementia, active psychosis);
- English-speaking.

Patients were excluded if actively suicidal or were unable to participate in a group setting (e.g., actively using substances, exhibiting threatening behavior).

### Study Arms

There were two study arms: **1)** TAU and **2)** SEGT.

**1)** The TAU group received standard care while admitted to hospital, which included care from an interdisciplinary team of rehabilitation professionals. The average length of stay for patients is four weeks, during which they are cared for by a team of nurses and hospitalists and are fitted and trained to use their prosthetics/orthotics under the supervision of prosthetists, occupational therapists, physiotherapists and a physical medicine and rehabilitation physician (physiatrist). Additional services from Social Work and Nutrition Services are also provided where required. Any TAU participants who required psychiatric care were also provided with mental health support from a psychiatrist who was not part of the study team.
**2)** SEGT was previously designed for patients with potentially life-threatening illnesses, including HIV and cancer patients, and has demonstrated efficacy in facilitating adjustment and coping while decreasing psychological distress in these populations.^16–18^ This SEGT program, usually delivered longitudinally in an outpatient setting, was adapted for LEA inpatients to enable and encourage participants to openly express and manage illness/disability-related emotions, increase social support, enhance relationships, improve symptoms and enhance body image. The treatment aims to facilitate mutual support and discussion of issues that are uppermost in patients' minds rather than imposing pre-determined topics for discussion.

For this study, the SEGT group participated in six one-hour sessions, held twice weekly over a three-week period. The course of treatment and number of sessions were designed to align with the average length of stay for LEA inpatients. The sessions were co-delivered by a psychiatrist and occupational therapist, and were framed within social cognitive theory^19^ whereby resilience to adversity (limb loss in this instance) relies on personal enablement.^20^ Please see **Appendix A** for a high-level summary of the salient topics raised by participants during the SEGT sessions. These topics closely align with the main foci or goals of the SEGT model previously defined in the literature.^21^

After the intervention, patients were provided with a patientfocused resource booklet created by the study team, which included resources on topics such as phantom pain, community support groups and coping.

### Primary Outcomes

The primary outcomes were on metrics related to the feasibility of implementing an inpatient SEGT trial for dysvascular LEA, which included:

1. Participant recruitment, retention, and follow-up rates;
2. Treatment adherence;
3. Survey completion rates (target set at 70%);
4. Completion rates of one-month post-intervention interview (target set at 50%).

### Secondary Outcomes

With regard to the secondary goal of determining the effectiveness of SEGT, the following surveys were administered to all participants in the study.

- Coping Self-Efficacy Scale (CSES) measures perceived self-efficacy for coping with challenges and threats.^22^
- Hospital Anxiety and Depression Scale (HADS) is a 14-item depression and anxiety screening tool that asks participants to rank the severity of their depression and anxiety symptoms.^23^
- Short Form-36 Survey (SF-36) is the most widely used health-related quality of life tool.^24^ The tool measures eight domains related to social, physical and mental health.
- Amputee Body Image Scale-Revised (ABIS-R) is a measure of body image perception in people living with limb loss.^25^
- Reintegration to Normal Living Index (RNLI) assesses involvement in recreational and social activities perceived ability to move within the community, and the degree of comfort people have with their relationships.^26^

Qualitative interviews with the SEGT participants and a focus group with healthcare providers working on the Amputee unit were undertaken to better understand which factors may have influenced study feasibility, and the potential impact of SEGT on wellbeing and quality of life post-LEA. Specifically, SEGT participants were asked to describe what they liked or disliked about the intervention, what benefits (if any) were obtained by taking part, and any recommendations for improvement. Providers were asked about their views of the potential impact of SEGT for patient care and its feasibility within inpatient settings.

### Procedure

Within three days of admission, all eligible LEA inpatients were approached by a member of their care team to determine if they were interested in participating. Patients who expressed interest in the study were then referred to the research team and a research coordinator obtained informed consent.

Data were collected on patients’ socio-demographics and impairment and all participants were approached to complete a battery of surveys (CSES, HADS, SF-36, and ABIS-R), within the first week of admission, at 24-72 hours post-discharge or SEGT completion, and at three months post-discharge. At three months post-discharge, participants also completed the RNLI. All participants were provided with a $25 gift card for participation.

Near the end of the study, the time window to collect discharge surveys was expanded to enhance the ability to capture additional data, as well as to inform our goal of informing the development of a future pragmatic trial. This protocol change was initiated due to challenges with contacting participants within 72 hours and other logistical issues (e.g., staff turnover). This change led to some of the discharge assessments (n = 7) being completed one month post-discharge, instead of the 72 hours outlined in the protocol.

One month after discharge, SEGT participants were also invited to complete the semi-structured interview. Interviews were conducted over the phone or over Zoom and took approximately 30 minutes (See **Appendix B** for the Interview Guide). Additionally, staff were invited to participate in an in-person focus group about their perspectives on SEGT, which took approximately 45 minutes. Both the interviews and focus group were recorded and transcribed for analysis.

### Sample Size and Analysis

Since this was a feasibility trial, a large sample size was not needed to adequately power statistical null hypothesis testing. A sample of 25 per group was deemed sufficient for the present trial as sample size of 12 per group is generally accepted as being sufficient for a pilot study.^27^

Descriptive statistics were used to evaluate the primary outcome measures. For the secondary outcome measures, a statistician who was blinded to group allocation conducted the analyses using t-tests. Participants with more than 20% missing data on a survey were excluded from analysis. For the qualitative interview and focus group data, the investigation team did not see the interview data until the trial ended to minimize bias. A narrative research approach^28–30^ was used to summarize the key insights by patients with LEA and clinical staff regarding the SEGT intervention. A study team member used an open coding framework to identify core issues related to perceived benefits and implementation considerations.

## RESULTS

The study was initiated in October 2021 and ended in February 2023; with 35 patients with dysvascular LEA identified as potential participants. Ten patients declined to participate because they were not interested (n = 8), not comfortable in participating (n = 1) or had a clinical suspicion of a cognitive impairment (n = 1). In total, 25 patients (mean age: 64.6 years; range: 42–79 years) agreed to participate, with 13 being randomized to TAU and 12 to SEGT. Eighteen of the participants were male and seven were female, with the majority of participants (n = 18; 72%) identifying as White (North American or European). The leading cause for the LEA was diabetes (n = 13; 52%), and most participants underwent a unilateral below the knee amputation (n = 16; 64%). **Table 1** presents the sample characteristics by study group.

**Table 1:** Socio-demographic and impairment characteristics.

Demographics		SEGT (n = 12)	TAU (n = 13)
Age, mean (SD)		65.1 (10.5)	64.1 (9.7)
Gender, n (%)	Female	3 (25.0)	4 (30.8)
	Male	9 (75.0)	9 (69.2)
Cause of Amputation, n (%)	Diabetes	8 (66.7)	5 (38.5)
	Peripheral Vascular Disease	3 (25.0)	3 (23.1)
	Ischemia/Embolism	1 (8.3)	4 (30.8)
	Other	0	1 (7.7)
Level of Amputation, n (%)	Below Knee (BKA)	8 (66.7)	8 (61.5)
	Above Knee (AKA)	4 (33.3)	2 (15.4)
	Bilateral AKA	0	2 (15.4)
	Bilateral BKA	0	1 (7.7)
Racial Group, n (%)	White (North American or European)	8 (66.7)	10 (76.9)
	South Asian	2 (16.7)	1 (7.7)
	Black-Caribbean	1 (8.3)	1 (7.7)
	Mixed Heritage	0	1 (7.7)
	Hispanic	1 (8.3)	0
Living Situation, n (%)	Living Alone	4 (33.3)	5 (38.5)
	Living with Others	8 (66.7)	8 (61.5)
Education, n (%)	High school or less	4 (33.3)	6 (46.2)
	Greater than High School	8 (66.7)	7 (53.8)
Employment, n (%)	Working	1 (8.3)	2 (15.4)
	Not working	11 (91.7)	11 (84.6)
Marital Status, n (%)	Married	6 (50.0)	6 (46.2)
	Not Married	6 (50.0)	7 (53.8)

**Figure 1:**
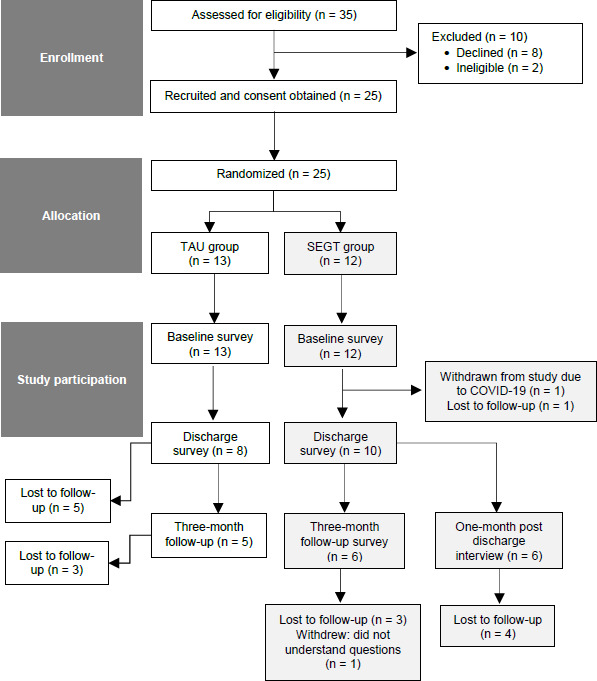
CONSORT flow diagram.

Of the 25 participants who consented, 21 participants completed the entire set of surveys for the baseline assessments and four participants (two in TAU; two in SEGT) only completed a portion of the baseline assessments. Following baseline surveys, one participant in the SEGT group was withdrawn due to COVID-19 precautions. For the discharge assessments, 18 participants were successfully contacted; 16 (eight in SEGT; eight in TAU) fully completed their discharge assessments and two TAU group participants partially completed the discharge assessments. For the three-month follow-up, 11 participants were successfully contacted, and completed the full set of assessments. Overall, 11 participants (five in TAU and six in SEGT) completed the full course of the surveys (see CONSORT diagram for more details). The survey completion rates were 84% for the baseline assessment, 64% for the discharge assessment, and 44% for the three-month follow-up assessment. The CONSORT flow diagram is described in **Figure 1**.

For the SEGT intervention, the mean number of sessions attended was 3.9 sessions (SD = 2.1). Four participants attended all six sessions, two attended five sessions, two attended three sessions, three attended two sessions, and one was withdrawn due to COVID-19 before attending any sessions. The main reasons for missed sessions included COVID-19 related absences (including illness and lockdowns), patients being transferred to different hospitals for medical reasons, and family visits. In total, only one SEGT participant completed the full course of the study, including all the SEGT sessions, surveys and the interview.

Due to the low survey completion rates at the three-month follow-up, t-tests were conducted to examine only baseline and exit data. The analysis of the secondary outcomes found no statistical significance between groups except on the HADS, whereby the SEGT group showed a significant improvement (p = 0.02; see **Table 2**). It should be noted that the ABIS-R had a high number of missing values as a result of most participants leaving more than half the items blank, which did not allow for an analysis of this outcome measure. As well, there were no significant differences in RNLI scores between the groups (SEGT M = 16.7; TAU M = 17.2), which was only collected at the three-month follow-up.

**Table 2:** Mean change scores on secondary outcomes and p-values for CSES, HADS, SF-36, and ABIS-R.

Outcome	Group	N	Baseline/Exit Change Score (SD)	P-value
CSES	SEGT	10	−7.46	0.37
	TAU	8	8.94	
HADS (depression)	SEGT	9	−0.11	0.74
	TAU	8	0.38	
HADS (anxiety)	SEGT	9	−4.39	0.02*
	TAU	8	−0.75	
SF-36 PCS	SEGT	23	30.05	0.97
	TAU	24	30.13	
SF-36 MCS	SEGT	23	56.12	0.16
	TAU	24	59.95	
ABIS-R	SEGT	N/A	N/A	N/A
	TAU	N/A	N/A	

**CSES:** Coping Self-Efficacy Scale; **HADS:** Hospital Anxiety and Depression Scale; **SF-36 PCS:** Short-Form 36 Physical Component Summary; **SF-36 MCS:** Short-Form 36 Mental Component Summary; **ABIS-R:** Amputee Body Image Scale-Revised; **SEGT:** Supportive-Expressive Group Therapy; **TAU:** Treatment as Usual; **N/A:** Not available

* Significant difference.

For the qualitative component of the trial, which was only offered to the SEGT group, six participants completed their one-month post-discharge semi-structured interview. All of the interviewed participants indicated they enjoyed participating in the group because it allowed them to socialize and form social connections with peers. For instance, one participant (ID#5022 [Male, age 63]) noted: *“I think the sessions created an opportunity to open up and embrace other people, and learn about other people, and understanding that basically we're all here on a journey and we're going through things that we can all relate to.”*

Many of the patients reported they experienced benefits from the sessions, such as getting out of their rooms, and hearing about the experiences of their peers. Regardless of the reason, participants reflected on how the group enabled them to increase comfort with disclosing their emotions.

*“I think I might cry, but you guys taught me. If you'd ask me a year ago, would I share what I'm feeling, I would've said no. I'd keep it in, I wouldn't tell anybody, and I would work it out in my own head…And I think, thinking back, all those guys in that room, it made me realize I can be vulnerable and I can open up”* ID#5008 (Male, aged 57).

Additionally, the topics discussed during the sessions were viewed by most participants as offering information that was useful and relevant to their lives. Even in cases when people did not feel the information was applicable to them personally, the group stimulated opportunities for personal reflection and mutual support.

In terms of recommendations, participants indicated they would have liked additional sessions (e.g. more than six) and to have larger group sizes. In particular, having someone with lived experience with limb loss who is further along in their recovery journey to join the group and share their post-discharge experience was seen as an important factor for gaining insight into adaptive coping. One participant (ID#5008 [Male, age 57]) suggested: *“I would've changed just one thing. I would've had a former patient in there the entire way, and just let them interject when they felt they needed to with the group. I think that would've helped me a lot, just to hear the truth… I want to hear that when I go home, I could hit a brick wall, I could do this, this could happen, this could happen.”*

As well, participants also requested additional educational resources outlining what their recovery and discharge may look like.

For the focus group, seven clinical staff took part, which included an occupational therapist (n = 1), nurses (n = 2), and physiotherapists (n = 4). Staff noted that a large number of patients are admitted with comorbid mental health and psychosocial challenges and that there is not enough mental health/psychosocial support offered to them. For instance, one staff (ID#PA2) commented: *“They usually have a lot of underlying mental health, psychosocial issues to begin with before the amputation. A lot of them, actually, in the lower socioeconomic status situation before they come. So, in terms of the support of mental health, they need a lot of practical support in terms of housing, funding for equipment, funding for transportation.”*

Importantly, they expressed concerns about the lack of mental health follow-up once patients were discharged into the community due to difficulty accessing community resources.

As for feasibility feedback for the group sessions, staff suggested that sessions occur in the afternoon to minimize conflict with their physical therapy and that more sessions be offered per week to increase opportunities for patient socialization. Staff also suggested implementing a system for patients to continue with sessions during outbreaks (e.g., use of tablet devices to move the group online when needed). Echoing the desires that the LEA participants expressed, staff suggested increasing the total number of sessions and providing more resources and educational materials on limb loss and recovery. Overall, the staff felt the group sessions were helpful and suggested continuing with them in an outpatient capacity to support the patients when they are discharged.

## DISCUSSION

The primary aim of this study was to collect feasibility data for an inpatient SEGT trial designed to help support the mental health needs of rehabilitation inpatients with LEA. Although in normal circumstances the feasibility of this study may be questioned, its completion during the COVID-19 pandemic with moderate rates of recruitment and session attendance, along with moderate to low survey completion rates, suggested that a larger trial would be feasible but should be modified to address some of the implementation challenges we encountered throughout the course of the trial (e.g., expanding the time frame to collect discharge data). This conclusion is supported by the qualitative feedback provided by participants and staff, while also accounting for contextual factors (e.g., COVID-19) in relation to feasibility outcomes.^31^ For instance, while the initial target sample size was 50 participants (25 for each group), several challenges primarily related to the COVID-19 pandemic (e.g., outbreaks and work from home policies) led to the recruitment of only 25 participants. This situation was not unique as several studies globally were disrupted by the pandemic.^32,33^ It should be noted that almost all of the participants referred to the study met the inclusion criteria, which indicates that the parameters set for participation were well-suited for this population. Without a pandemic, recruitment would likely have been more robust. Other feasibility trials of psychosocial interventions in individuals with LEA have reported a wide range of recruitment rates, from as low as 19% to as high as 79%.^34–37^ However, additional research is needed to establish appropriate recruitment and survey completion rates when evaluating the feasibility of delivering a group intervention to an inpatient dysvascular LEA population.

Regarding the study assessments, the a priori target for survey completion was set at 70%. This target was exceeded at the baseline assessment (84%) but not met at study exit (64%) or at the three-month follow-up (44%). The moderate rates of completion for discharge and the low rates at the three-month follow-up were attributed to research staff not being able to contact participants just prior to or following their discharge. For a future trial, loss to follow-up could be mitigated by extending the time-window to contact participants after discharge from three days to one week (as patients are frequently engaged with other pre- and post-discharge appointments). Additionally, the outcome measure related to body image (ABIS-R) had high rates of missing data, which suggests participants may have found this measure difficult to complete. Body image issues post-limb loss are well-documented,^1,7^ and group therapy has been shown to help persons cope,^15^ but further work is needed to determine if the lack of response to the ABIS-R was related to participants’ not wanting to reflect on this topic or factors associated with the measure itself.

With regard to group session attendance rates for the SEGT group, the average attendance was 3.9 sessions (SD = 2.1), with 50% attending five or more sessions. Again, the main factor for low attendance was related to factors associated with the COVID-19 pandemic. For example, the second SEGT group cohort was cancelled after three sessions due to an outbreak on the inpatient unit. For future studies conducted during any type of outbreak, one recommendation is to offer participants virtual sessions if in-person sessions are not feasible. There is some evidence suggesting that it is feasible to switch from in-person to virtual SEGT but further work is needed to establish and validate best practices.^38,39^

The secondary measures were largely inconclusive except for a larger change score in depression and anxiety by the SEGT group compared to the TAU condition; suggesting that taking part in SEGT may lead to an improvement in mood, which aligns with the findings of other group therapy interventions for persons with disabilities^40^, as well as for individuals with limb loss.^15^

While promising, it is possible that this finding is due to chance as a result of the lack of power from having a small sample size and missing data; all of which limits the generalizability of this finding. There may also be a dose effect, which can only be measured if the SEGT intervention was extended beyond the initial six sessions. An adequately powered pragmatic trial would help determine the effectiveness of a delivering an SEGT intervention for dysvascular LEA.

Participants with LEA and clinicians also offered several suggestions on ways to improve the feasibility and acceptability of the SEGT intervention. This included adding more sessions, delivering the group later in the day, increasing group size, adding more educational material as well as having overlapping cohorts, whereby newly admitted patients could interact with those closer to discharge.

There are several limitations with the present study. First, the COVID-19 pandemic created a number of operational disruptions in terms of recruitment, delivery of the SEGT intervention, and follow-up with patients regarding discharge assessments. Although not a primary objective, the survey data related to the secondary outcomes is inconclusive, with only the depression and anxiety scales showing some initial promise. Due to a protocol adjustment toward the end of the study, some of the participants completed their discharge assessments a few days after discharge, while others completed them a month after discharge. This discrepancy may have affected survey scores given that some participants had an extended time of being at home. Due to the large degree of missing data at follow-up, examining potential confounders was not possible. As such, we cannot conclusively determine the clinical benefits of SEGT over standard care, but qualitative data provides some insights about the therapeutic value of SEGT. Importantly, while our sample had some participants from diverse backgrounds, the majority were White and English-speaking, and further work is required to explore SEGT's applicability for participants with different cultural attitudes, expectations and needs.

Finally, the study protocol was relatively well-adhered to, although some deviations did occur due to the pandemic. To account for these unexpected challenges for future trials, it would be beneficial to extend the initial recruitment windows and the timeframe for survey completion. Despite these limitations, critical insights have been obtained to inform the development of a pragmatic trial, which is currently being pursued (ClinicalTrials.gov ID: NCT05798091) and that has incorporated some of the suggested modifications derived from our qualitative investigations (i.e., open–ended recruitment, continuous weekly sessions). The outcomes of the pragmatic trial will provide additional evidence on whether SEGT is a clinically valid approach for this population.

## CONCLUSION

This study provides evidence for the feasibility of a SEGT intervention, a form of group therapy, for inpatients with dysvascular LEA undergoing rehabilitation. These findings provide a number of important insights on how to better implement a SEGT program for LEA inpatients, with some qualitative data outlining its perceived benefits. Further work is needed to determine the clinical validity of SEGT for LEA, and to further explore implementation considerations to optimize its’ delivery.

## Appendix A

**ID# Date:**   **DD**/MM/YYYY

**Patient Interview Guide**

Thank you for agreeing to participate in an interview. For this interview, we are hoping to gain a better understanding of your experience with your participation in the research study testing a new group therapy approach for inpatients with limb loss at St. John's Rehab. The questions listed below are intended to guide our conversation and may vary slightly depending on how that conversation unfolds.

Throughout the interview, please feel free to share as much or as little as you feel comfortable with.

***To start off, I want to learn a little bit about who you are and your initial experiences with losing your limb(s).***

1. Please tell me a little more about where you are from, where you live, your family, etc.?
2. Please tell me what led to you needing to have your limb(s) amputated?
3. What were you feeling or thinking about before you had to undergo the surgery for your amputation?
  - What were your top concerns or needs?
  - How did healthcare providers meet those needs/concerns?

***Now I want to focus on your experience at St. John's Rehab for your limb loss rehabilitation care.***

4. Could you tell me what happened when you arrived at St. John's Rehab?
  - What happened when you arrived?
  - How were you feeling?
  - What was helpful during this time?
  - What made things more difficult?
5. What types of mental health or social supports did you receive?
  - Were you seen by a social worker or psychiatrist? Why were you referred to them?

***These questions will focus on your experience of taking part in the psychosocial group at St. John's Rehab.***

6. When you were first approached to take part in the study, what were your thoughts about participating in a group therapy?
  - Why were you interested in taking part?
  - What concerns, if any, did you have about it?
  - What were you expecting it to look like?
7. Please describe the types of activities you did as part of the group?
  - How many sessions did you participate in? If you missed any, why was that?
8. What were the most beneficial aspects of taking part in the group?
  - What do you feel went well? Why?
  - Were your expectations met?
  - Why do you think this aspect was beneficial?
9. What were the least beneficial aspects of taking part in the group?
  - What aspects of the program could have been improved? Why?
  - Were there any aspects you feel could be removed from the program? Why?
  - Was there something the program was missing that you'd like to see added?
10. What were your top concerns and/or needs while taking part in the group?
  - Were these concerns/needs met during your time in the program? If not, why do you feel they were not met?
  - How did healthcare providers meet those needs/concerns? If not, how could they have better supported you?

***These last set of questions will focus on your experience of transitioning back to home from rehab.***

11. Prior to leaving St. John's Rehab, what were your top needs or concerns?
  - Did you feel prepared to leave?
  - How did healthcare providers meet those needs/concerns? If not, how could they have better supported you?
12. Since you've left the hospital, how do you feel you are adjusting to being back at home and in your community?
  - What has gone well with getting back home?
  - What has not gone as expected or well? How have you managed or dealt with things that have not gone as planned
13. Has there been anything from the group therapy program that you have applied to help you adjust to being back at home?
  - If so, what have you used?
  - If not, what do don't you think you haven't used it?

Thank you for taking the time to share your experiences. This brings us to the end of the interview questions. Is there anything else you'd like to share about your experiences taking part in the therapy group or recovery that we haven't touched on? *[If yes, let participant discuss. If no, reiterate our gratitude for their time and participation]*

## Appendix B:

Themes Discussed During SEGT

**Table:**

Coping mechanisms used to deal with limb loss	Participants often discussed personal coping strategies to deal with their amputation, including humor, positivity, praying, smoking, and writing. Acceptance of limb loss is adaptive, and can be considered as a stage of grief. Trying to maintain control can be adaptive or maladaptive. Relying on others for support can help with coping, but relying too much on others can drive them away (maladaptive). It is important to find a purpose in life and to let this guide your mindset (i.e. yoga, being athletic) Importance of mindfulness and meditation as a positive coping strategy.
Camaraderie with peers/peer support	Participants in the group created a “brotherhood” and united in their stories of limb loss. Described finding solace and support in others who have also experienced limb loss. During one of the sessions, each participant showed their residual limb to the group, and compared the size of wheelchairs, surgical scars, phantom pain etc. Peer comparison of experiences from acute care to rehab helps with perspective and recovery.
Adaptation after limb loss	Discussions about how adaptable the participants are. The idea of having to “move past the standard”— doing things differently to enjoy the same things as everyone else (i.e. driving).
The importance of self-advocacy	Discussion surrounding the importance of advocating for yourself during recovery. Participants encouraged each other to ask questions and encourage help-seeking. Self-advocacy can get you better care and more choices, but there is a need to ask first. Rather than depending on people to offer support, there is a growing realization that with an amputation, one may need to show initiative to ASK for support.
Attitudes towards healthcare and clinical care	Frustration around the healthcare system. Some good experience with staff, but also many discussions about frustration with the skills and perceived lack of transparency of some healthcare providers. Loss of faith in the healthcare system, feelings that the healthcare system has let them down. Difficulties with navigating the healthcare system alone and a lack of guidance from professionals. Wanting to be informed but feel like providers are not being transparent.
The importance of family & support	Family and friends often support amputees during their entire journey, from discussions leading up to the amputation, their recovery post-surgery, and preparing the hope (renovations). How families often act as advocates for patients. Family as motivator–wanting to get better for family. The “family” also includes friends who call and offer support.
Relationships	Worries about how their amputation affects their families, which includes the trauma that families are experiencing. Friends are also struggling with the participant's amputations. Talk about how to discuss limb loss with children in the family with the hope of normalizing the experience. They also want spouses and family to have a support group, so they can better understand what the patient is experiencing.
Pain	Discussions around physical pain and managing pain medications. The idea of getting used to pain, especially chronic pain or phantom pain. The connection between pain and mood — people can sometimes get irritable when in pain. Bonding over emotional pain — and how to manage it. Phantom pain was an important topic discussed, including the differing severity of symptoms and the use of mirror therapy to treat it. Most patients in the group experience it, and it is really severe.
The importance of having meaning & purpose during recovery.	Participants desire to be occupied and to have a purpose. Try to stay occupied during their recovery. Feelings of helplessness and uselessness expressed throughout the recovery. Pride in having hobbies (building decks, car models). Create a routine so it feels more like home.
Setbacks during recovery	Discussions about procedures and medical complications that came up during their recovery. Not feeling happy with recovery due to continued pain.
Progress during recovery	Gaining confidence and independence with improved mobility and the use of a wheelchair. Growing acceptance of their limb loss and how that changes their perspective. Even small progress is a big win. Progress improves self-confidence and dignity.
Loss & grief	Grieving lost abilities as a result of amputation and having to adapt to a “new normal” (things they can no longer do) Having to rely completely on other people for care is a loss of dignity. Feelings around being completely helpless. Loss of independence. Loss of driving license, independence and past self. Coming to terms with all of the things you can no longer do.
Covid difficulties	Frustrations around isolation, restrictions, and delays. Missed groups due to COVID restrictions/infections.
Faith	Having faith in Sunnybrook as a teaching hospital. The idea of karma and questioning God. Feeling punished by God. Loss of faith due to limb loss.
Gratitude	Participants discussed how grateful they were for recovering both physically and emotionally, and for social support. Grateful for having hope. How grateful they are for the experiences at the hospital including helpful staff and supportive peers that encourage recovery. Participants were happy knowing their own injury was not worse.
Worries about the future	Most participants agreed that they are anxious about what will happen when they are discharged. Dealing with practical day-to-day challenges and worries around handling finances. Being unprepared for discharge due to having an inaccessible home.
Overall feedback on group	Individuals felt it was “informative” and a “nice spot” as “people listen here”. Helped them build relationships with one another, despite differences in background, future Overall, it allowed them to learn how to relate to one another. Groups might have facilitated acceptance of one's condition. Allowed people to open up and speak even though they normally would not have.
